# Dental Laws of North Dakota

**Published:** 1890-06

**Authors:** 


### Dental Law of North Dakota.

An Act to Revise and Amend an Act Entitled "An Act to Insure the Better Education of Practitioners of Dental Surgery, and to Regulate the Practice of Dentistry in the Territory of Dakota."

*Be it enacted by the Legislative Assembly of the State of North Dakota:*

SECTION 1. That it shall be unlawful for any person who is not, at the time of the passage of this act, lawfully entitled to practice dentistry in this State, pursuant to the provisions of the act of which this act is a revision and amendment, to practice or attempt to practice dentistry in this State, unless such persons shall have first received a license to practice dentistry from the board of dental examiners, as hereinafter provided.

SEC. 2. A board of examiners to consist of five practicing dentists is hereby created, whose duty it shall be to carry out the purposes and enforce the provisions of this act. The members of said board shall be appointed by the governor. The term for which the members of said board shall hold their offices shall be five years, except that the members of the board appointed by the governor pursuant to the provisions of the act to which this is an amendment, residing in North Dakota, and acting as such at the time this act shall take effect, shall hold their respective offices until the close of the term for which they were respectively appointed. The offices of those living in South Dakota are hereby declared vacant and shall be filled as in case of any other vacancy. Any vacancy in said board, whether by removal, death, resignation, or otherwise, shall be filled by the governor No person who shall be in any manner pecuniarily interested in, or who shall be officially connected with, any dental college or dental department of any school or university, shall be appointed a member of said board.

SEC. 3. Said board shall have power to make reasonable rules and regulations for carrying into effect and maintaining the provisions of this act. It shall choose one of its members president, and one secretary thereof, and shall hold regular meetings twice in each year, and such other regular and special meetings as said board may by its rules provide. A majority of said board shall at all times constitute a quorum thereof for the transaction of business, but a less number may adjourn from time to time. The board shall keep full and complete minutes of its proceedings, and of its receipts and disbursments, and a full and accurate list of all persons licensed and registered by said board, and such records, together with the list of licensed and registered dentists, to be kept as aforesaid, shall be public records, and shall at all reasonable times be open to public inspection, and such records, or a transcript of the same, or of any part thereof, under the seal of the board, duly certified by the secretary thereof, shall at all times and places be competent evidence of the facts therein stated or recited. A sworn statement by the secretary, under the seal of the board, stating that any person is or is not a registered dentist, shall be *prima facie* evidence that such person is or is not entitled to practice dentistry in this State. The president of the board and the secretary thereof shall have authority to administer oaths, and the board shall have power to hear testimony as to all matters relating to the duties imposed upon it by law.

SEC. 4. It shall be the duty of every person who, at the time this act shall take effect, is a legally qualified practitioner of dentistry in this State, as shown by the books of registration kept by said board, under the provisions of the act of which this is an amendment, and who is desirous to continue such practice, and of all persons who shall thereafter be licensed by said board to practice dentistry, to procure form the secretary of said board, on or before the thirty-first day of May, 1890, and annually thereafter a certificate of registration as a practitioner of dentistry in this State. Such certificate shall be issued by the secretary upon payment of a registration fee, to be fixed by the board, which fee shall not exceed the sum of two dollars. All certificates so issued shall expire on the thirty-first day of May in each year, and shall be *prima facie* evidence of the right of the holder thereof to practice dentistry in this State during the time for which they were issued. Any certificate or license granted by said board may be revoked by the board, upon conviction of the party holding it, of a violation of any of the provisions of this act. Every person receiving such certificate shall conspicuously expose the same in his place of business.

Sec. 5. Any person having pursued the study of dentistry in the office, or under the supervision of some regularly practicing dentist, for at least three years before applying for such examination, not lawfully entitled to practice dentistry at the time when this act shall take effect, who shall thereafter desire to practice dentistry in this State, shall appear before said board and be examined with reference to his knowledge and skill in dentistry, and if, upon such examination, such person be found, in the judgment of said board, to possess suitable qualifications to practice dentistry, and if the board shall be satisfied that the applicant has a good moral character, it shall issue to such person a license to practice dentistry in accordance with the provisions of this act. *Provided*, That any person desiring to commence the practice of dentistry in this State, and having a diploma issued or purporting to be issued by any reputable dental college, or dental department of any university, shall present the same to the State board of examiners, and said board being satisfied as to the genuineness of the diploma, and the qualifications of the applicant, shall issue a license to such person to practice dentistry in this State without examination, on payment of the license fee hereinafter provided for. All licenses issued by said board shall be signed by the several members thereof, and be attested by its president and secretary, and the seal of said board.

Sec. 6. Any member of said board may issue a temporary license to any applicant upon the presentation of such applicant of satisfactory evidence that he possesses the necessary qualifications to practice dentistry, on the payment of ten dollars, which license shall remain in force until the semi-annual meeting of said board occurring next thereafter, and no longer; but such license shall not be renewed, nor shall it be granted to any applicant who has, within six months previous to his application, been rejected by said board. Such license shall not be valid until it shall be attested by the secretary of the board, under its seal, and the secretary shall keep a record of such licenses, the date of their issue, and the name of the members by whom each license was issued.

SEC. 7. Any person shall be regarded as practicing dentistry within the meaning of this act, who shall perform upon the human teeth, or parts adjacent thereto, any operation or operations such as are commonly known and designated as dental operations, or operations in dental surgery; or who shall hold himself or herself out, by means of signs, cards, advertisements, or otherwise, as a dentist or dental surgeon. Any legally qualified practitioner of dentistry who has complied with the provisions of this act, or any properly organized and equipped and reputable dental college, or dental department of any reputable school or university, may take into preceptorship a student or students who shall be permitted to perform such operations in the offices or infirmaries of such preceptors and under their immediate supervision, and not otherwise, during the term of three years from the commencement of such pnpilage, and no longer, unless for special reasons such time shall, in the discretion of the board, be thereafter extended for a period not exceeding one year. *Provided*, nothing in this act shall be construed to prevent any legally qualified resident physician and surgeon from extracting teeth, or to prevent any person from using any domestic remedy or other proper means for the relief of pain in case of an emergency.

SEC. 8. In order to provide means for carrying into effect and maintaining the provisions of this act, said board of dental examiners may require each person appearing before it for examination as aforesaid to pay said board a fee not exceeding ten dollars, which shall in no case be returned to such applicant; and if the applicant shall receive a license to practice, he shall thereupon pay the further sum of five dollars, which shall entitle him to receive also a certificate of registration as a practitioner of dentistry in this State for the current or registration year in which such license shall be issued, after the termination of which he shall annually obtain a certificate as hereinbefore provided. All moneys received by the board shall be held by the secretary thereof as a special fund for paying the necessary expenses and the compensation of the board and its secretary, as herein provided, and for enforcing the provisions of this act; and the secretary shall give such bond as the board may from time to time require. No part of the salaries or other expenses of the board shall be paid out of the State treasury, but the annual report of the board shall be printed by the State. The secretary of the board shall receive a salary which shall be fixed by the board, in addition to the necessary and legitimate expenses by him incurred in the discharge of his duties, and each member of the board shall receive as compensation the sum of five dollars per day for each day actually employed by him in attending meetings, or in performing any special duty assigned to him by the board, and shall be reimbursed for all legitimate and necessary expenses by him incurred in the performance of any official duty. Said board shall, on or before the first day of December in each year, make an annual report of its acts and proceedings to the governor, which report shall contain, among other things, an accurate statement of all moneys received and disbursed during the previous year.

SEC. 9. Any violations of any of the provisions of this act shall subject the party violating the same to a penalty of not less than twenty-five dollars nor more than fifty dollars for the first offense; of not less than fifty dollars nor more than one hundred dollars for the second offense, and not less than one hundred dollars nor more than two hundred and fifty dollars for the third or any subsequent offense, and such penalties shall be sued for and recovered in any court of competent jurisdiction in the name of the people by the State's attorney of the county wherein such offense shall have been committed, or in which the offender may be found, and said penalty, when recovered, shall be paid into the common school fund of the county in which the suit shall be brought; and in case of the non-payment of such penalty, the party so offending shall be liable to imprisonment for a period not exceeding six months, in the discretion of the court having cognizance thereof. *Provided*, that either party may appeal in the same time and manner as appeals may be taken in other cases, except that where an appeal is prayed in behalf of the people, no appeal bond shall be required to be filed, whether the appeal be from a justice of the peace, or from the county or district court, or from the appellate court. But it shall be sufficient in behalf of the people of the State of North Dakota, for the use of the board of dental examiners, to pray an appeal, and thereupon an appeal may be had without bond or security. *Provided*, further, That no proceeding shall be commenced against any party for failure to procure the annual certificate of registration provided for in Section 4, until after such party shall have been served with proper notice of such failure, and the penalty thereby incurred. Each operation performed and each patient treated, contrary to the provisions of this act, shall be deemed and held as a separate offense.

SEC. 10. Any person who shall willfully and falsely claim or pretend to have or hold a certificate of license or registration of this board, or of any similar board of any other State, or who shall willfully and falsely, with intent to deceive the public, claim or pretend to be a graduate of, or hold a diploma granted by any incorporated dental society or dental college, shall be subject to the penalties provided for in Section 9 of this act, to be sued for and recovered and paid out as in said section provided.

SEC. 11. All laws or parts of laws in conflict with this act are hereby repealed.

Approved February 6, 1890.

---

NEW METHOD OF EXAMINING NERVOUS TISSUES IN A FRESH STATE.—Kronthal (*Neurologisches Centralblatt*, No. 2, 1890), has described a method for examining, in a fresh state, the microscopical characters of the central nervous system. A piece—about as big as a pin's head—of brain or spinal cord, as the case may be, should be taken quite fresh and placed upon the object-glass. It is then covered with a cover-glass and pressed out flat. A drop of a 0.5 per cent. solution of methyl blue is placed at the edge of the cover-glass, which is raised to let the stain run in. After from thirty seconds to a minute the superfluous stain is removed with blotting-paper. The cover-glass is then raised; the preparation is allowed to dry in the air. This takes five or ten minutes. A drop of Canada balsam is then added, and the preparation is ready for examination.—*British Medical Journal.*

